# Unsuccessful external validation of the MAC-score for predicting increased MIB-1 index in patients with spinal meningiomas

**DOI:** 10.3389/fonc.2022.1037495

**Published:** 2022-11-29

**Authors:** Victor Gabriel El-Hajj, Alexander Fletcher-Sandersjöö, Jenny Pettersson-Segerlind, Erik Edström, Adrian Elmi-Terander

**Affiliations:** ^1^ Department of Clinical Neuroscience, Karolinska Institute, Stockholm, Sweden; ^2^ Stockholm Spine Center, Löwenströmska Hospital, Upplands-Väsby, Stockholm, Sweden

**Keywords:** MIB-1 (Ki-67 labeling) index, score, spinal meningioma, proliferation, clinical implications, recurrence, external validation

## Abstract

**Objective:**

Recently, the MAC-spinal meningioma score (MAC-score) was proposed to preoperatively identify spinal meningioma patients with high MIB-1 indices. Risk factors were age ≥ 65 years, a modified McCormick score (mMCs) ≥ 2, and absence of tumor calcification. The aim of this study was to externally validate the MAC-score in an independent cohort.

**Methods:**

Using the same inclusion and exclusion criteria as in the original study, we performed a retrospective, single-center, population-based, cohort study that included patients who had undergone surgical treatment for spinal meningiomas between 2005 – 2017. Data was collected from patient charts and radiographic images. Validation was performed by applying the MAC-score to our cohort and evaluating the area under the receiver operating characteristic curve (AUC).

**Results:**

In total, 108 patients were included. Baseline and outcome data were comparable to the original development study. An increased MIB-1 index (≥5%) was observed in 56 (52%) patients. AUC of the MAC-score in our validation cohort was 0.61 (95% CI: 0.51 – 0.71), which corresponds to a poor discriminative ability.

**Conclusion:**

The MAC-score showed poor discriminative ability for MIB-1 index prediction in patients with spinal meningiomas. Moreover, the MAC-score rests on a weak theoretical and statistical foundation. Consequently, we argue against its clinical implementation.

## Introduction

Spinal meningiomas are intradural extramedullary tumors that originate from the arachnoid cap cells in the leptomeninges of the spinal canal. They are the most common adult primary spinal tumor, accounting for 25-45% of all spinal intradural tumors and occurring with an age-adjusted incidence of 0.33 per 100, 000 population (1).

Even though most spinal meningiomas are benign (World Health Organization (WHO) grade 1), (2) they can cause spinal cord compression and neurological deficits. (3) Surgery is the treatment of choice for symptomatic patients, (2) and often associated with improved neurological function. (4) The functional status in these patients is usually assessed using standardized methods, such as the modified McCormick scale (mMCS) (**Table 1**). (4–6) Tumor proliferation markers, like the MIB-1 index, are also often used to assess the growth fraction of the tumor cells. Although previous studies are scarce, spinal meningiomas tend to have low MIB-1 indices (7–9) and there is no consensus on a specific MIB-1 index cut-off value for the prediction of tumor progression or recurrence in spinal meningiomas.

**Table 1:** Modified McCormick scale.

Grade	Explanation
1	Intact neurologically, normal ambulation, minimal dysesthesia
2	Mild motor or sensory deficit, functional independence
3	Moderate deficit, limitation of function, independent w/external aid
4	Severe motor or sensory deficit, limited function, dependent
5	Paraplegia or quadriplegia, even w/flickering movement

Wach et al. recently developed a risk score to preoperatively predict a high MIB-1 Index (≥ 5%) in these patients. (10) The MAC-spinal meningioma score awards 1 point each for Age ≥ 65 and preoperative mMCs ≥ 2, and 2 points for the lack of intra-tumoral calcification. They reported an area under the receiver operating characteristic curve (AUC) of 0.83 (95% CI: 0.71 – 0.96) in their development cohort and concluded that the score could help support surgical decision making (10).

The performance of risk scores is typically overestimated in the datasets used to develop them. (11–14) They are often opportunistically produced to maximize the output from a study for which the tested predictors were not declared beforehand. Therefore, risk scores should always be subjected to external validation in an independent cohort. (13, 14) In the case of the MAC-score, it was developed from single-center data on 128 patients, with no internal validation or pre-published study protocol.

In light of the above, the aim of this study was to perform an external validation of the MAC-score in an independent cohort of adult patients who were surgically treated for a spinal meningioma.

## Methods

### Patient selection and study setting

**Figure 1:**
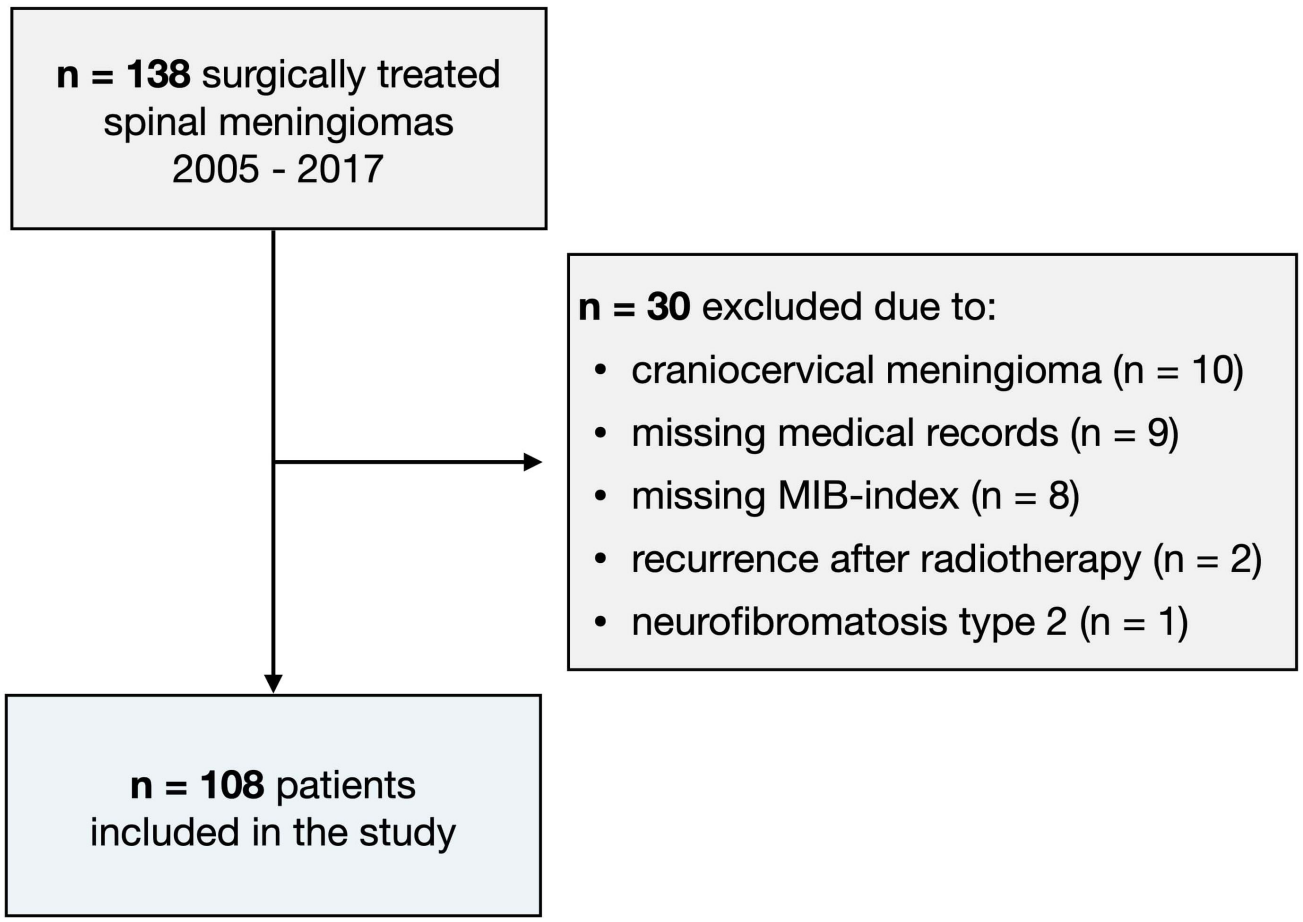
Flow-chart illustrating the patient inclusion process.

The study cohort consisted of adult patients (≥18 years) who were surgically treated for a spinal meningioma at the study center between 2005 and 2017. Exclusion criteria were identical to those in the development study, (10) namely craniocervical meningiomas (foramen magnum, C1, C2), neurofibromatosis type 2 (NF2), recurrent meningiomas after radiotherapy, and those with missing MIB-1 index (**Figure 1**). The study center’s routine for preoperative imaging, surgical technique, and follow-up has been described previously. (3, 4) The study was approved by the Regional and National Ethical Review Board who waived the need for informed consent (Dnr: 2016/1708-31/4 and 2020-00192).

### Variables

Medical records and imaging data were retrospectively reviewed using the health record software TakeCare (CompuGroup Medical Sweden AB, Farsta, Sweden). Collected data included age, sex, preoperative modified McCormick Scale (mMCs), radiographic data (including tumor calcification and location), surgical data, MIB-1 index and World Health Organization (WHO) grade, as well as long-term tumor control and functional outcome.

In accordance with the study by Wach et al, (10) age was dichotomized into elderly (≥ 65 years) and non-elderly (18–64 years). Tumor calcification was assessed on preoperative CT and/or MRI images by two different reviewers. (9, 15) A tumor was deemed calcified when it was hyperintense on CT, reflecting a density close to that of adjacent bony structures, or when the tumor had decreased signal intensity on T1 and T2. (9, 16) Tumor growth was defined as the radiological growth of a tumor remnant following subtotal resection, while tumor recurrence was defined as the appearance of a new spinal meningioma following total resection. All histopathological analyses were performed at the Department of Pathology, Karolinska University Hospital, Stockholm, Sweden. The MIB-1 labeling index was determined using the anti-Ki67 antibody (product: “M7240, Ki-67 Antigen”; supplier: DAKO, Glostrup, Denmark). The MIB-1 labeling index was then analyzed by experienced pathologists at the authors’ institution through manual counting of the number of Ki-67 positive cells (only nuclear staining) divided by the total amount of tumor cells in “hot-spot” regions, counting at least 2000 cells. Patients were classified according to WHO criteria from 2007. However, as no patients showed signs of spinal cord invasion, the grading is consistent with the 2016 WHO classification of meningiomas (17, 18).

### Statistical analysis

The Shapiro–Wilk test was used to evaluate the normality of the data. As all continuous data significantly deviated from a normal distribution pattern (Shapiro–Wilk test p-value < 0.05), it is presented using the median (interquartile range) and categorical data as numbers (proportion). Demographics, clinical data, and imaging data were stratified by MIB-1 index and compared using the chi-square test for categorical data and the Mann–Whitney U test for continuous variables. In addition, a uni- and multivariable logistic regression analysis was used with the MAC-score components as explanatory variables and MIB-1 index ≥ 5% as the binary outcome. Lastly, discrimination of the score was quantified by calculating the area under the receiver operating characteristics curve (AUC) statistic. Generally, an AUC value of 0.9 – 1.0 represents excellent, 0.8 – 0.9 good, 0.7 – 0.8 fair, and 0.6 – 0.7 poor discriminative ability. (19) The prognostic validity of the MAC-score was further investigated by calculating the sensitivity and specificity of each threshold. All statistical analyses were carried out in R (version 4.1.2). Statistical significance was set at p < 0.05.

## Results

### Patient characteristics

Of the 138 patients screened, 108 were included in the study (**Figure 1**). Complete data for all risk factors (mMCs ≥ 2, age ≥ 65 years, tumor calcification) were available in all included patients. The median age was 66 years (IQR 56 – 73) and 89 (82%) were female. Fifty-six patients (52%) had a MIB-1 index ≥ 5%. The median pre-operative mMCs was 2 (IQR 2 – 3), and the most common tumor location was the thoracic spine (n = 81, 75%). Fifteen (14%) of the tumors were calcified, and the median MIB-1 index was 5 (IQR 3 – 5). One-hundred and seven (99%) of the tumors were WHO grade 1, and one tumor was grade 2 (0.9%) (**Table 2**).

**Table 2:** Patient characteristics.

Variable	Patients (n = 108)
Age (years)	66 (56 – 73)
Female sex	89 (82%)
Pre-operative mMCs	2 (2 – 3)
Calcified tumor	15 (14%)
MIB-1 index	5.0 (3.0 – 5.0)
Tumor location	
Cervical	26 (24%)
Thoracic	81 (75%)
Lumbar	1 (0.9%)
Simpson grade	
Simpson grade II	78 (72%)
Simpson grade III & IV	30 (28%)
WHO grade	
WHO grade 1	107 (99%)
WHO grade 2	1 (0.9%)
Tumor growth or recurrence	4 (3.7%)

Data is presented as median (interquartile range) or number (proportion). mMCs, modified McCormick scale; WHO, World Health Organization.

### Association between MIB-1 index and clinical features

Univariable and multivariable associations between MIB-index ≥ 5% and baseline characteristics, imaging, and surgical data in the validation cohort, including the three components of the MAC-score, showed significant association only for tumor calcification (p = 0.008), but not for mMCs ≥ 2, age ≥ 65 years, sex, tumor level, tumor extent, anterior tumor location, or Simpson grade (**Tables 3**, **4**).

**Table 3:** Data comparison between patients with a normal (< 5%) and increased (≥ 5%) MIB-1 index.

Variable	MIB-1 < 5% (n = 52)	MIB-1 ≥ 5% (n = 56)	p-value
Age (years)	66 (55 – 74)	66 (58 – 72)	0.973
Female sex	41 (79%)	48 (86%)	0.349
Preoperative mMCs	2 (2 – 3)	2 (2 – 3)	0.479
Calcified tumor	12 (23%)	3 (5.4%)	**0.008**
Cervical tumor	14 (27%)	12 (21%)	0.434
Anterior tumor component	10 (19%)	14 (25%)	0.471
> 2 spinal segments	4 (7.7%)	5 (8.9%)	>0.999
Simpson grade III & IV	18 (35%)	12 (21%)	0.126

Bold text in the p-value column indicates a statistically significant correlation (p < 0.05). Data is presented as median (interquartile range) or number (proportion).mMCs, modified McCormick scale.

**Table 4:** Univariable and forced-entry multivariable logistic regression analysis predicting MIB-1 index ≥ 5%.

Variable	OR (95% CI)	Univariablep-value	Multivariable p-value
mMCs ≥ 2	1.23 (0.49 – 3.13)	0.663	0.721
Age ≥ 65 years	1.14 (0.53 – 2.45)	0.731	0.496
Absence of tumor calcification	5.30 (1.56 – 24)	**0.014**	**0.012**

mMCs, modified McCormick scale.

Bold text in the p-value column indicates a statistically significant correlation (p < 0.05).

### External validation of the MAC-score

**Figure 2:**
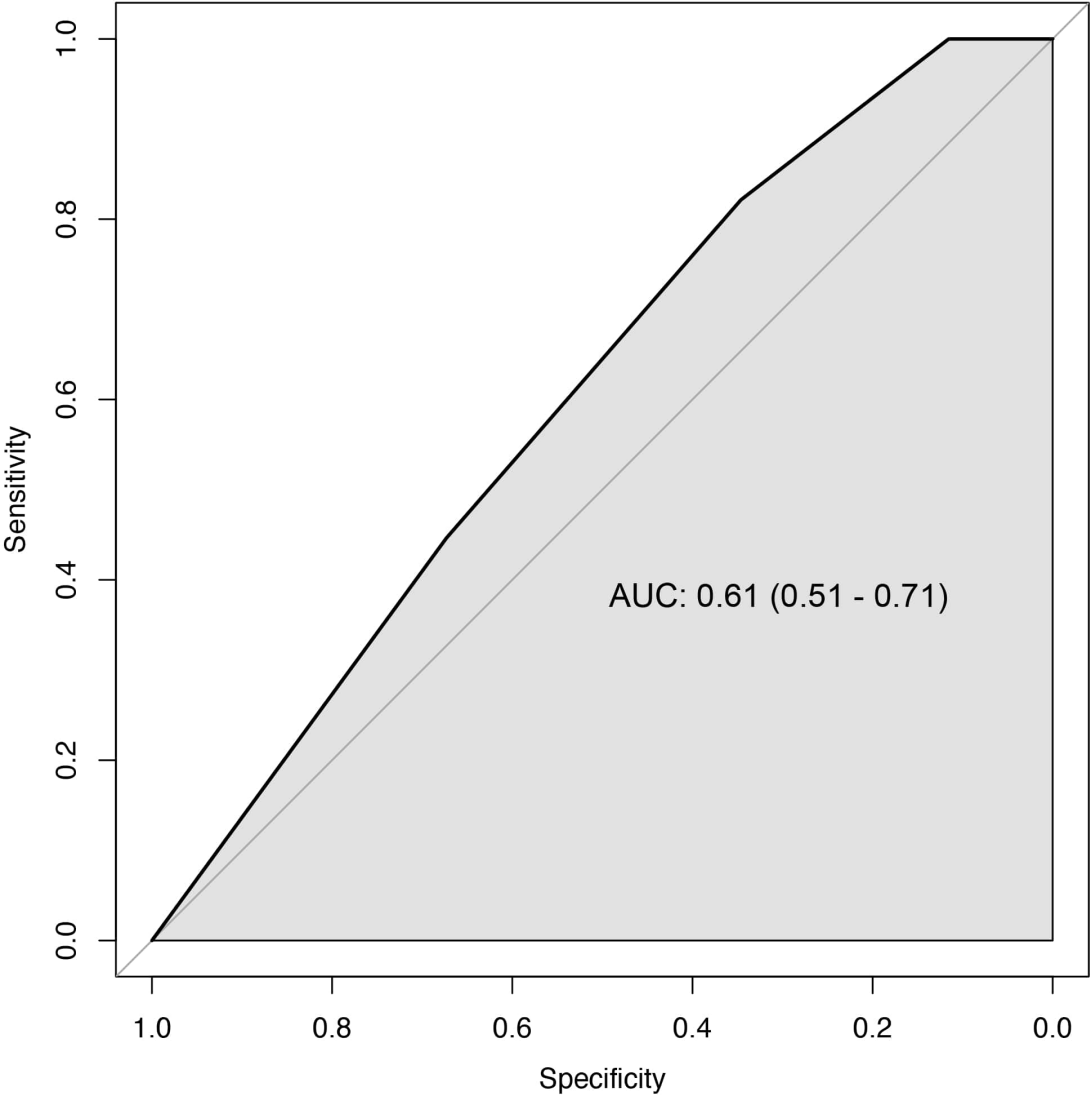
ROC curve of the MAC-scores ability to identify patients with high MIB-1 index (black line: AUC 0.61, 95% CI 0.51 – 0.71). The diagonal grey line indicates the model that has a completely random discrimination power.

In our validation cohort, the AUC for the MAC-spinal meningioma score was 0.61 (95% CI: 0.51 – 0.71) (**Figure 2**). The cut-off points of 1, 2, 3 and 4 showed a sensitivity of 100%, 100%, 82%, and 45%, and a specificity of 2%, 12%, 35%, and 67%, respectively (**Table 5**). These results imply poor discriminative ability of the score in our cohort.

**Table 5:** Sensitivity and specificity for different MAC-score thresholds.

MAC-score threshold	Sensitivity	Specificity
MAC 1	100%	2%
MAC 2	100%	12%
MAC 3	82%	35%
MAC 4	45%	67%

## Discussion

### Principal findings

We sought to externally validate the recently proposed MAC-score for preoperative prediction of high MIB-1-index in patients with spinal meningiomas. (10) The score awards two points for the lack of calcification, and one point each for higher age (≥ 65) and poor preoperative mMCs (≥ 2). A higher MAC-score was suggested to indicate an increased risk of MIB-1 index of ≥5% and be able to discriminate between stable and growing spinal meningiomas. The original study also suggested a correlation between MAC-score and longer hospital stay as well as increased likelihood of improved postoperative mMCs. In our validation cohort, the AUC for the MAC-spinal meningioma score was 0.61 (95% CI: 0.51 – 0.71), as compared to 0.83 (95% CI: 0.71 – 0.96) in the original development cohort. Thus, the score showed poor discriminative ability for MIB-1 index prediction in this independent cohort. To further examine this failed validation of the MAC-score, its main parameters will be discussed below.

### Effect of calcification

Tumor calcification was a significant predictor of low MIB-1 index in our study. This is in line with the study by Wach et al. and with the body of evidence regarding calcification as a marker of reduced growth potential of meningiomas. (20) This is also consistent with data from intracranial meningiomas. (20) The calcified appearance of spinal meningiomas on CT is thought to represent tightly packed psammoma bodies or the formation of metaplastic lamellar bone microscopically (17, 18). With varying definitions, calcified spinal meningiomas have been reported to make up 2.6 to 75% of the total. (5, 21, 22) While calcified spinal meningiomas may represent more quiescent tumors, they are associated with more surgical complications and a less favorable functional outcome after surgery, especially when ossification is found intraoperatively (23–27).

### Effect of age

We could not verify the finding that older age was significantly associated with higher MIB-1 index. Previously published evidence rather seems to indicate that young age is associated with an increased risk of recurrence. (28–31) Notably, several studies have also failed to find any significant correlation between age and tumor recurrence. (5, 7, 21, 32) Previous studies also indicate that elderly patients benefit from surgery for spinal meningiomas and there are no significant differences regarding extent of surgery, complications, or recurrence. (4, 5, 7, 33, 34) Studies on the correlation between age and MIB-1-index in cranial meningiomas have failed to show significant results. A large study on 1372 patients found a nonsignificant trend towards higher MIB-1 index in older patients, (35) and another study on 385 patients, showed no differences in MIB-1 indices in relation to age. (36) Moreover, there is no evidence pointing towards spinal meningiomas having a more aggressive behavior in elderly patients. (4, 37).

### Effect of preoperative mMCs

We found no association between a higher mMCs score and a higher MIB-1 index, thereby contradicting the findings by Wach et al. (10) Arguably, fast growing tumors may result in more severe neurological deficits and higher preoperative mMCs. However, there is currently no evidence to support the argument that a MIB-1 index ≥ 5% accelerates tumor growth sufficiently to negatively impact preoperative functional status. The vast majority of spinal meningiomas have indices lower than 4% (7, 37, 38) and further studies are needed to clarify the clinical utility of the MIB-1 index for the predominantly low-grade spinal meningiomas. Wach et al. also reported that patients with a higher MAC-score improved more than those with a lower score at three months follow-up. (10) Since the MAC-score partly rests on the mMCs data, and only patients with preoperative symptoms can improve, this finding becomes self-evident. In addition, two previously published studies found that the improvement in mMCs was correlated to the degree of spinal cord compression rather than MIB-1 index, (4, 6) and age, sex, tumor location, and MIB-1 index all failed to significantly correlate with postoperative mMCs improvement. (4).

### Hospital stay

Wach et al. also explored the association between MIB-1 index and length of hospital stay, suggesting that MIB-1 index ≥ 5% was associated with longer hospital stay. However, they present no hypothesis as to why meningioma patients with a MIB-1 index ≥ 5% would require a longer hospital stay. In our experience, length of hospital stay reflects local referral structures between surgical clinics and rehab centers as much as actual clinical aspects. Regarding the latter, more complex surgeries, complications, (16, 39) and the management of patients with comorbidities are likely to result in prolonged hospital stay. Conversely, as argued above, the evidence suggests that calcified tumors with a low MIB-1 index, rather than tumors with a high MIB-1 index, are associated with more surgical complications and longer hospital stays (22, 26, 27).

### MIB-1 index

The same methodology was used to determine the MIB-1 index in this validation study and in the study performed by Wach et al, indicating negligible variability in the measurement of MIB-1 indices.

Regardless, inter-observer and inter-laboratory variabilities in the measurement of proliferation indices like the MIB-1 index have previously been reported. (40–43) However, the resulting errors are systematic rather than random and affect all measurements performed in a given laboratory and by a given pathologist in a similar manner. While the absolute numbers and averages would differ between analyses performed in different settings, (40) the relative distributions would not, as an element of proportionality should remain. This implies that associations with the MIB-1 index, when treated as a continuous variable, should be preserved in the presence of a big enough sample. However, when dichotomizing MIB-1 indices, as performed in the study by Wach et al, an absolute cut-off value determined at one laboratory may not be valid at another. In accordance, several studies have shown that MIB-1 cut-off values suggested for the prognostication of tumors have limited reproducibility between centers in a multi-center setting. (40, 41) This, in turn, limits the generalizability and usability of the MAC-score.

### Methodological aspects

This validation study has several methodological strengths in relation to the development study by Wach et al. The same MIB-1 index determination technique, inclusion and exclusion criteria were used and the distribution of sex, age, tumor calcification, tumor location, pre-operative mMCs, MIB-1 indices, and the rate of tumor recurrence were similar. (4) The validation cohort was population-based, with few exclusions due to missing data thus minimizing selection biases.

It is likely that the unsuccessful validation of the MAC-score is partly due to type I errors in the original study. For instance, Wach et al. performed multiple comparisons on the same dependent variable without compensating for the number of inferences made. This could have been performed using a Bonferroni correction. Alternatively, the authors could have limited the events per variable (EVP), defined as the number of events divided by the number of predictor variables used. An EVP of 10 is often advocated as a minimal criterion in logistic regression analyses. (11) For the study by Wach et al. where 55 events and 19 predictors where identified, an EVP of 2.9 was calculated. This level is associated with considerable risk for type I errors. (44) Furthermore, as no pre-hoc statistical analysis plan was published, the steps leading to the choice of the evaluated parameters making up the MAC-score cannot be evaluated. In addition, the development study should ideally have randomly divided the cohort into a derivation and validation subset, allowing for internal validation to avoid overfitting. It should also be noted that the authors presented mMCs using means and standard deviations, even though it is an ordinal variable and should have been presented using medians or proportions at different cut-offs.

### Clinical remarks

In the study by Wach et al, a higher MAC-score indicated an increased likelihood of elevated MIB-1 index. Because a higher MIB-1 index was significantly associated with a higher recurrence rate, the authors deduced that the MAC-score was also a predictor of tumor progression and recurrence rate. However, there were only four recurrences in the material and progression was not studied. Furthermore, the authors identified a correlation between MAC-score and the length of hospital stay without providing an explanatory theory for this. The authors concluded that the score may “support preoperative patient-surgeon consultation, surgical decision making and enable a tailored follow-up schedule”. However, gross total resection is the gold standard for treatment of spinal meningiomas, and we question the clinical usefulness of pre-operative prediction of MIB-1 index in surgical decision making, as compared to radiographic findings and clinical presentations. It has also been demonstrated that shorter time from diagnosis to surgery is a predictor of postoperative improvement, advising against a watch-and-wait strategy. (4) In the postoperative phase, it will be the extent of tumor resection, findings of the histopathological analysis, and clinical status of the patient that decide the management plan.

To assist clinicians in the management of spinal meningioma patients, a clinical score or biomarker needs the power to accurately predict outcomes or risk of tumor recurrence. For outcome prediction, the score should ideally be based on factors available before surgery to allow an informed decision regarding whether surgery should be performed or not. In addition, the score should rely on prognostic factors with an established mechanistic role in the disease. Poor preoperative status, longer time to surgery and reoperation are all predictors of unfavorable outcome. (2, 4) Similarly, known risk factors for tumor recurrence may for example include higher WHO grade and higher Simpson grade resection. (2, 45, 46) Hence, in our opinion, large multicentric datasets are needed to yield enough power for scores to directly predict outcomes of clinical interest (unfavorable neurologic status or recurrence) relying on well documented and logical predictors.

## Conclusion

The MAC-spinal meningioma score showed poor discriminative ability when externally validated in an independent cohort. Gross total resection is the gold standard treatment of spinal meningiomas, and a pre-operative prediction of MIB-1 index will have little to no impact on surgical decision making. Based on these findings, clinical implementation of the MAC-spinal meningioma score is discouraged.

## Data availability statement

The raw data supporting the conclusions of this article will be made available by the authors, without undue reservation.

## Ethics statement

The studies involving human participants were reviewed and approved by Regional and National Ethical Review Board. Written informed consent for participation was not required for this study in accordance with the national legislation and the institutional requirements.

## Author contributions

Data acquisition: VE-H, AF-S, JP-S and AE-T. Statistical analysis: AF-S. Data interpretation: VE-H, AF-S, JP-S, EE, and AE-T. Writing and creation of tables and figures: VE-H, AF-S, AE-T. Proof reading: EE. All authors approved of the final version prior to submission.

## Funding

This research was supported by Region Stockholm FoUI-959989.

## Conflict of interest

AE-T was supported by Region Stockholm in a clinical research appointment.

The remaining authors declare that the research was conducted in the absence of any commercial or financial relationships that could be construed as a potential conflict of interest.

## Publisher’s note

All claims expressed in this article are solely those of the authors and do not necessarily represent those of their affiliated organizations, or those of the publisher, the editors and the reviewers. Any product that may be evaluated in this article, or claim that may be made by its manufacturer, is not guaranteed or endorsed by the publisher.
